# Macrophage Polarization and Liver Ischemia-Reperfusion Injury

**DOI:** 10.7150/ijms.52691

**Published:** 2021-01-01

**Authors:** Hai Wang, Zhifeng Xi, Lu Deng, Yixiao Pan, Kang He, Qiang Xia

**Affiliations:** Department of Liver Surgery, Renji Hospital, School of Medicine, Shanghai Jiao Tong University, Shanghai, China.

**Keywords:** liver, ischemia-reperfusion injury, macrophage polarization

## Abstract

Ischemia-reperfusion injury refers to organ damage caused by the previous insufficient supply of oxygen and nutrients and the involvement of metabolic by-products after blood flow is restored. Liver ischemia-reperfusion injury (IRI) has become a hot research in recent years, because it occurs in many clinical scenarios. After the introduction of liver transplantation and vascular control techniques in liver surgery, liver ischemia-reperfusion injury is considered to be an important factor affecting postoperative mortality and morbidity. As the largest immune organ in the human body, liver contain a lot of immune cells such as resident macrophages (Kupffer cells), dendritic cells, natural killer cells, and natural killer T cells which play a key role in ischemia-reperfusion injury. Among those, macrophage-mediated excessive inflammatory response is considered to be an important factor in liver ischemia-reperfusion injury. The prominent feature of liver injury is an increase in the number of macrophages in liver due to the infiltration of blood monocytes and differentiation into monocyte-derived macrophages. Liver macrophages can be divided into M1 macrophages which can promote inflammation progress and M2 macrophages that inhibit inflammation progress according to their different phenotypes and functions. Both of them can regulate liver aseptic inflammation, and play an important role in triggering, maintaining, and improving liver ischemia-reperfusion injury. This review summarizes studies of macrophage polarization on liver ischemia-reperfusion injury in recent years, to provide potential ideas for translation application in future clinical management.

## Introduction

Liver ischemia-reperfusion injury (IRI) has become a hot research topic in recent years, because this condition occurs in many clinical settings, such as hemorrhagic shock and resuscitation, trauma, liver resection, and liver transplantation 1. After the introduction of vascular control techniques in liver surgery, liver Ischemia reperfusion injury has become an important factor affecting postoperative mortality and morbidity 2. Liver dysfunction caused by liver ischemia-reperfusion injury can further affect remote organs and cause systemic damage 3.

Liver ischemia-reperfusion injury is divided into the initial and late stages. The early manifestation of IRI is the rapid activation of KCs after reperfusion, and the late IRI is characterized by the recruitment of neutrophils to the liver after ischemia 4.

As the largest immune organ in the human body, the liver contains many immune cells such as resident macrophages (Kupffer cells; KCs), dendritic cells (DCs), natural killer (NK) cells, and natural killer T (NKT) cells which play key roles in ischemia-reperfusion injury 5.

There are many different types of immune cells in the liver, and an excessive liver macrophage-mediated inflammatory response is considered to be an important factor in liver ischemia-reperfusion injury. Liver macrophages can be divided according to their different phenotypes and functions into M1 macrophages which can promote inflammation progression and M2 macrophages, which inhibit inflammation progression. Both phenotypes can regulate aseptic liver inflammation, and play an important role in triggering, maintaining, and improving liver ischemia-reperfusion injury 6.

In this review, we aimed to summarize the mechanism of liver ischemia-reperfusion injury, the polarization of liver macrophages, and the effects of these cells on liver ischemia-reperfusion injury.

## Hepatic ischemia-reperfusion injury (IRI) and liver macrophages

Ischemic organ injury refers to an oxygen shortage in the organ due to insufficient blood supply, and can disrupt cell metabolism. Ischemia-reperfusion injury refers to organ damage caused by insufficient supply of oxygen and nutrients and the involvement of metabolic byproducts after blood flow is restored^4^. IRI mainly manifests as swelling of endothelial cells and KCs, vasoconstriction, leukocyte infiltration and platelet aggregation, and eventually leads to blockage of the microcirculation. Activated KCs and neutrophils exacerbate this process by secreting inflammatory factors and free radicals 1. Hepatic ischemia-reperfusion injury occurs in many clinical settings, such as liver resection, liver transplantation, and trauma. Vascular occlusion technology is often used to avoid excessive bleeding during liver surgery, and understanding the mechanism of liver ischemia-reperfusion injury is beneficial in the clinical care of many patients 4.

The two main types of liver injury due to ischemia-reperfusion are distinct 7. Warm IRI is caused by liver cell damage occurs situ during liver transplantation or in response to various forms of shock or trauma, and may cause liver or even multiple organ failure. Cold IRI is caused by damage to hepatic sinusoidal endothelial cells and destruction of the microcirculation, which occurs during storage in vitro and is usually accompanied by warm IRI during liver transplantation. Although these two different types of liver injury have different initial cellular targets, they share similar characteristics, such as activation of liver KCs and neutrophils, production of cytokines and chemokines, generation of ROS, increased expression of adhesion molecules and infiltration by circulating lymphocytes and/or monocytes 8.

Liver ischemia-reperfusion injury can also be divided into initial and late stages. The early manifestation of IRI is the rapid activation of KCs after reperfusion, and the late IRI is characterized by the recruitment of neutrophils to the liver after ischemia 4.

The initial stage of liver ischemia-reperfusion injury occurs within minutes and up to six hours after liver ischemia. This stage is mainly due to metabolic disorders caused by hypoxia, and oxygen free radicals are generated that cause liver damage. At this stage, damaged cells produce reactive oxygen species (ROS) by producing intracellular xanthine oxidase and NADPH oxidase 9, and cell swelling due to dysfunction of the cellular sodium-potassium pump 10. ROS can activate liver KCs to produce more ROS and cytokines. ROS produced by KCs are the main source of damage to the liver and cells, but xanthine oxidase and NADPH oxidase inside liver cells also contribute to ROS production 11. These ROS cause cellular oxidative stress and damage to liver parenchyma and blood vessels. Although the degree of liver ischemia-reperfusion injury at this stage is modest, it causes a series of advanced injuries, including the production of a series of proinflammatory factors that recruit activated white blood cells and cause significant liver damage 3, 4. Pathogen-associated molecular patterns (PAMPs) produced by exogenous pathogens and danger-associated molecular patterns (DAMPs) released by necrotic cells activate Toll-like receptors (TLRs) to stimulate the immune response. Toll-like receptors can be found in KCs, dendritic cells, and liver cells. TLRs can induce cells to release proinflammatory factors such as TNF-α and IL-6 after stimulation. TNF-α stimulates hepatocytes and KCs to secrete neutrophil chemoattractants (such as CXC chemokines) and proinflammatory factors (such as TNF-α, IL-6), and causes upregulation of endothelial cell adhesion molecules such as ICAM-1, VCAM-1 and P-selectin to induce capture of blood neutrophils 4.

In the late stage of hepatic ischemia-reperfusion injury neutrophils are recruited to the liver after ischemia and damage liver cells by producing ROS and proteases. Hepatic ischemia-reperfusion injury is affected by not only cellular metabolism and oxygen free radicals in the late stage, but also by the inflammatory response. Liver damage is also more severe in the late stage than in the early stage. Endothelial cells can secrete chemokines to attract neutrophil adhesion. Recruited neutrophils and hepatocytes interact to activate NADPH oxidase or myeloperoxidase in neutrophils to produce ROS such as hydrogen peroxide (H2O2) or hypochlorous acid (HOCL). ROS diffuses into liver cells, which causes mitochondrial dysfunction and leads to imbalance of intracellular calcium ion concentration and causes apoptosis 12. In addition to producing ROS, neutrophils can also secrete proteases by exocytosis to destroy the basement membrane and extracellular matrix 13.

Many studies reported that T cells are indispensable in liver ischemia-reperfusion injury. Systemic immunosuppression (cyclosporine, FK506) reduces liver IRI, suggesting that T cells play a very important role 14. Circulating CD4 T lymphocytes can be stimulated by cytokines secreted by neutrophils that are recruited to the liver in late IRI, resulting in cell accumulation in the liver 15. They can also be suppressed by anti-inflammatory factors such as IL-10 16.

The liver contains the largest proportion of macrophages in the parenchymal organs. In healthy rodents, there are 20-40 macrophages per 100 hepatocytes. It can be seen that liver macrophages play a very important role in maintaining the homeostasis of the liver itself and the entire body 17. Liver macrophages can be divided into three sources: yolk sac, fetal liver and bone marrow. Macrophages in the liver can also be divided into KCs residing in liver tissue and blood monocyte-derived macrophages (MDMs). KCs are distributed along the hepatic sinusoidal endothelial cells and are able to continuously remove intestinal pathogens from the blood 18. Monocyte-derived macrophages mainly exist in the portal triad, and play a role in iron and cholesterol metabolism 19. KCs are derived from fetal liver-derived red bone marrow progenitor cells and yolk sac-derived red bone marrow progenitor cells that express macrophage colony stimulating factor 1 receptor and are self-renewing instead of relying on infiltrating monocytes to maintain themselves 20. Blood monocyte-derived macrophages are derived from bone marrow hematopoietic stem cells.

A prominent feature of liver injury is an increase in the number of liver macrophages. After liver inflammation occurs, the number of liver macrophages increases due to blood monocyte infiltration and differentiation into monocyte-derived macrophages. The differentiation, morphology and function of monocyte-derived macrophages are independent of KCs. As mentioned above, the maintenance of the number of Kupffer cells depends on self-renewal rather than monocyte infiltration. However, if KCs are depleted during liver injury, monocyte-derived macrophages can acquire the phenotype of KCs and can proliferate and differentiate into KCs 17.

KCs have a strong phagocytic ability in hepatic sinusoids, and can take up apoptotic cells and pathogens in the liver. KCs can also be stimulated by danger signals to release a series of inflammatory mediators such as reactive oxygen nitrogen substances, pro-inflammatory factors IL-1, IL-6, TNF-α, and chemokines to cause damage during liver ischemia-reperfusion. In addition, liver macrophages are closely related with the differentiation of hepatic progenitor cells, the regulation of liver cell lipid and carbohydrate metabolism, and a series of pathological conditions of the liver such as liver fibrosis and liver cancer 21.

## Macrophage polarization

Macrophages have the ability to alter their phenotype in response to changes in the surrounding microenvironment. The phenotypic variability of macrophages that affects the ability of macrophages to secrete a series of different cytokines 22. Under the influence of IFNs, the stimulation of Toll-like receptor ligands, and the cytokine such as IL-4/IL-13, macrophages will be activated and transformed into M1 or M2 types, which respectively represent two extremes on a continuous spectrum of macrophage activation states. The macrophage population will be biased towards M1 or M2 in vivo depending on physiological conditions (such as organ regeneration or pregnancy status) and pathological conditions (such as allergies, infections, and chronic inflammation). The coexistence of different activated macrophages can also be seen under specific clinical conditions. Understanding the mechanism of macrophage activation and transformation and the molecules involved are helpful for establish a series of clinical diagnosis and treatment 23.

Generally, macrophages are divided into M1 type, which promotes inflammation and M2 type that suppresses inflammation by the difference of secreted cytokines and cell surface markers.

M1 macrophages, also known as classically activated macrophages, differentiate from macrophages stimulated by IFN-γ or ligands of Toll-like receptors 22, 24. These macrophages are characterized by high expression of CD80, CD86, MHCII, Toll-like receptor 4 and inducible nitric oxide synthase 6. This macrophage has a strong ability to destroy microorganisms and tumor cells and can secrete high levels of proinflammatory cytokines such as IL-6, TNF-α, IL-1, IL-12, IL-15 and IL-18 etc^25^. In addition, M1 macrophages can also secrete chemokines such as CXCL9, CXCL10, CXCL11, CXCL15, and CXCL20 to recruit other immune cells, such as Th1 cells, and induce a severe Th1 immune response. At the same time, they can also act as innate immune effector cells and secrete reactive oxygen species (ROS) and nitric oxide (NO)^26^. IFN-γ can be produced by both innate immune cells and acquired immune cells. The representative of innate immune cells is NK cells, which can maintain early transient macrophage activation, and IFN-γ secreted by acquired immune cells (TH1 cells) is necessary to maintain long-term activation of macrophages. Activated macrophages increase cell killing by secreting superoxide anions and reactive oxygen and nitrogen species and promoting a Th1 immune response 27.

M2 cells, also known as alternatively activated macrophages, are differentiated from macrophages stimulated by IL4/13^23^. These macrophages are characterized by high expression of CD206, CD161, arginase, chitin 3-like protein and inflammatory local molecules 6. M2 macrophages play an important role in anti-inflammatory, and antiparasitic activities, promoting angiogenesis, promoting wound healing and fibrosis of extracellular matrix, and can also stimulate tumorigenesis. M2 cells can be divided into M2a, M2b type and M2c type. M2a macrophage differentiation occurs under the induction of IL-4 and IL-13, differentiate into M2b types under the induction of immune complexes and toll-like receptor ligands, and M2c differentiation occurs under the induction of IL10 and TNF-β^26^. Basophils and mast cells and other granulocytes are the main sources of early secretion of IL4, which induces macrophage differentiation 27. IL-4 can stimulate arginase expression in M2 macrophages. Activity, converts arginine to ornithine, which as a precursor of polyamines and collagen, promotes the production of extracellular matrix, thus promoting tissue regeneration and wound repair 28. M2 macrophages can secrete the anti-inflammatory factors IL-10, chemokines CCL22 and CCL17 to promote the recruitment and activation of Th2 cells to suppress excessive immune response 29. However, when M2 macrophages excessively promote the repair and reorganization of extracellular matrix, it can also cause damage. Studies have shown that tissue fibrosis in chronic schistosomiasis is caused by uncontrolled activation of M2 macrophages during wound healing. When inhibiting the IL-4 receptor on M2 macrophages or using antibodies against the IL-4 receptor can reduce the degree of fibrosis in the lesion 30 (Table 1).

Macrophages can be divided into the M1 type that promotes inflammation and M2 type that suppresses inflammation. However, in addition to liver inflammation, M1 and M2 macrophages also play important roles in other liver disease models. In liver cancer, tumor-associated macrophages (TAMs) play key role. Tumor-associated macrophages acquire the M2 phenotype after exposure to cytokines (such as IL-4 and IL-13) and signals secreted by tumor cells. M2 tumor-associated macrophages promote angiogenesis and lymphogenesis and suppress the anticancer immune response to promote liver cancer progress. On the contrary, Tumor-associated macrophages can differentiate into M1-type macrophages after being stimulated by LPS and IFN-γ to play a role in suppressing cancer 21. Depletion of NDRG2 (N-myc downstream-regulated gene 2) can shift tumor-promoting M2-like TAMs toward the M1-like phenotype can lead to tumor suppression via phagocytosis and the secretion of inhibitory factors 31. When M2 macrophages excessively promote the repair and reorganization of extracellular matrix, it can also cause liver fibrosis. Studies have shown that tissue fibrosis is caused by uncontrolled activation of M2 macrophages during wound healing. Inhibition of the IL-4 receptor on M2 macrophages or using antibodies against the IL-4 receptor, it can reduce the degree of fibrosis of the lesion 30. In addition, obesity and HCV and HBV infections in chronic liver disease are also thought to be related to liver macrophage polarization 17, 25.

## Polarization of M1 macrophages and liver ischemia-reperfusion injury

During hepatic ischemia-reperfusion, hypoxia increases liver ATP catabolism, leading to the accumulation of hypoxanthine, its breakdown product. Furthermore, hypoxia also leads to exhaustion of antioxidants, ischemia activates calcium-dependent harmless xanthine dehydrogenase, which is harmless and is converted to harmful xanthine oxidase, and a large amount of ROS are generated using intracellular molecular oxygen. These oxygen free radicals cause the imbalance of intracellular calcium ion concentrations and changes cellular pH, causing cell death and liver injury, and the release of DAMPs 6, 11. HMGB1 is one of the key endogenous DAMPs. HMGB1 is significantly up-regulated after 1 hour of ischemia-reperfusion. HMGB1 binding to TLR4 activates KCs to polarize to the M1 phenotype though the STAT1 pathway, release inflammatory factors such as TNF-α and further release ROS to promote liver damage 32. An increase in the number of M1-type macrophages can be observed in the tissues 8-72 hours after the occurrence of liver ischemia-reperfusion injury 27. In addition to HMGB1, other DAMPs such as histamine, DNA fragments, and ATP can also activate M1 polarization of KCs through different TLR. The downstream elements of the TLR4 signaling pathway include adaptor molecules, MyD88 and TRIF (TIR domain-containing adaptor inducing IFN-β). HMGB1 activates M1 polarization of macrophages through a TRIF-dependent signaling pathway, and activates M1 type macrophages to release proinflammatory factors such as TNF-α 4.

In addition to HMGB1, IFN-γ can activate macrophages through the JAK-STAT signaling pathway. IFN-γ activates JAK, resulting in STAT1 phosphorylation and the promotion of M1 polarization 33.

The Notch pathway mediated by RBP-J is also thought to be related to M1 macrophage polarization. Notch signaling pathway is a highly evolved and conservative path. The ligand Dll4 binds to the Notch1 receptor to form a complex that activates the downstream ADAM protease and regulates γ-secretase, causing NICD to enter the nucleus, interact with RBP-J, and finally promote M1 type polarization 33.

Epigenetics and noncoding mi-RNA also influence macrophage polarization. Specifically, miR-9, miR-127, miR-155 and miR-125b have been proven to induce M1 polarization while miR-124, miR-223, miR-34a, let-7c, miR-132, miR-146a and miR-125a-5p promote M2 macrophages by targeting different transcription factors and adaptor proteins 34. The clinical potential of non-coding Mi-RNA has gradually gained attention.

Long noncoding RNAs (lncRNAs) is also involved in polarization of macrophage. lncRNA-MM2P has been shown to activate M2 macrophage polarization and inhibit M1 macrophage polarization. Knocking out lncRNA-MM2P will reduce the phosphorylation of STAT6 to inhibit the effect of cytokines to promote M2 polarization of macrophages, and thereby reduce the role of M2 cells in promoting angiogenesis 35.

Activated M1 macrophages can exacerbate the inflammatory response by secreting a series of pro-inflammatory factors, of which TNF-α is a very important one. TNF-α can bind to TNF receptors on the surface of liver cells and activate NF-κB and c-Jun N-terminal kinase (JNK) pathways pathway to induce cell death and worsen liver damage 36. TNF-α can also induce tissue damage through the peroxisome proliferator-activated receptor-γ co-activator (PGC)-1α/mitofusion (Mfn)-2 pathway to affect mitochondrial function and cell metabolism 37. In addition, TNF-α can also trigger inflammatory responses by promoting macrophages to secrete other inflammatory factors such as ROS, and up-regulating endothelial cell adhesion molecules and inducing liver cells to express CXC chemokines. Furthermore, CD4 T cells were temporarily recruited to the liver. As the expression of CXC chemokines and adhesion molecules increases, neutrophils are also recruited to the liver parenchyma. Neutrophils directly damage liver cells by releasing ROS and proteases, resulting in cell necrosis and death 4. TNF-α can cause not only swelling of hepatic sinusoidal endothelial cells, but also cause liver microcirculation disturbance by neutrophils and endothelial cells recruited by the previously mentioned process 38.

DAMPs can also stimulate the formation of inflammasomes in M1 macrophages, thereby promoting inflammatory response. Inflammatory bodies are multimeric protein complexes assembled in the cytoplasm of macrophages, that consist of at least three proteins: activated NOD-like receptor (NLR), apoptosis-associated speck-like protein containing CARD (ASC) and pro-caspase 1. There are different NLR proteins associated with inflammasomes, of which the NLRP3 inflammasome is the most thoroughly documented. The formation of inflammatory bodies requires two signals. The first signal is pattern recognition receptors (PRRs) were stimulated by DAMPs and up-regulated transcription of pre-IL-1β and IL-18. The second signal includes lysosomal components, ATP, ROS, increased calcium ions, uric acid and other components. Stimulated by a second signal, pro-caspase 1 is self-cleaved in inflammasome to form caspase 1, and pro-IL-1β were cleaved into active IL-1β and released to the tissue 17. This process promotes the production of HMGB1 39 and recruiting neutrophils in the blood circulation^40^.

**Figure 1:**
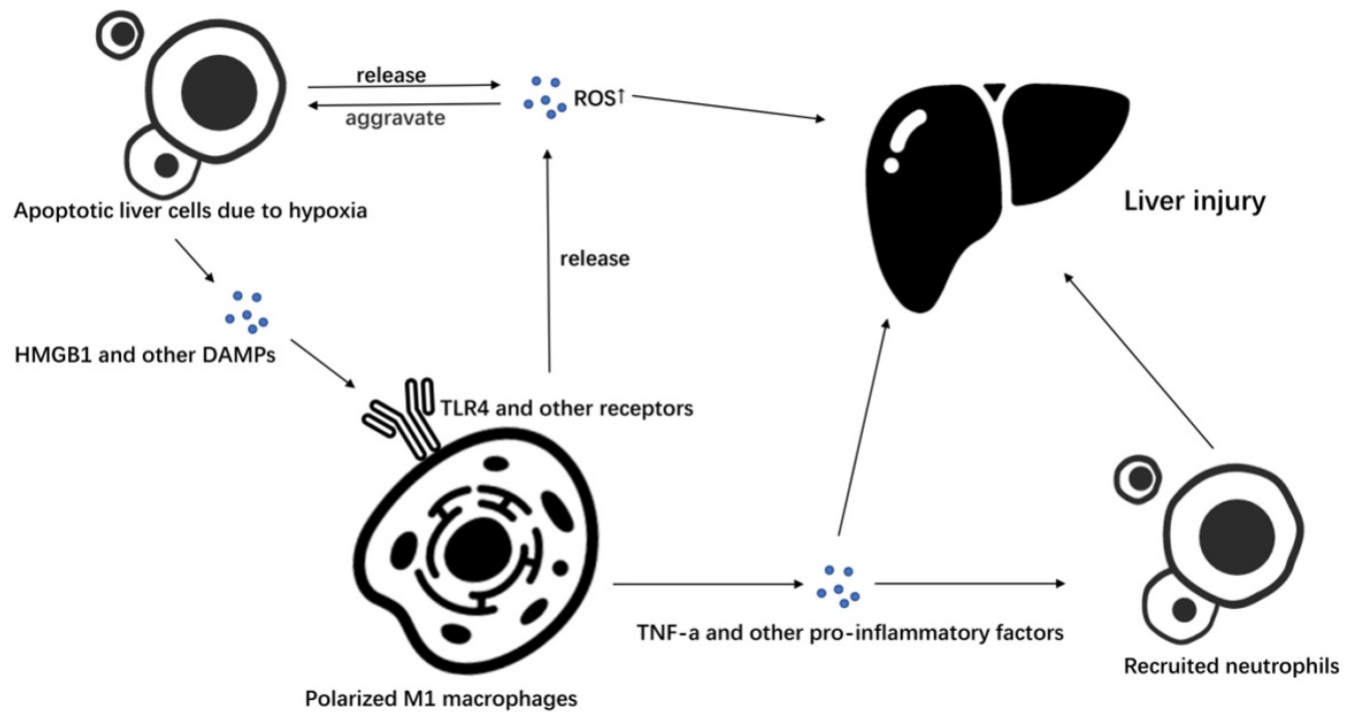
M1 macrophages in liver ischemia-reperfusion injury. In liver ischemia-reperfusion, liver cells produce a large amount of ROS due to hypoxia and apoptosis occurs. Apoptotic liver cells secrete a series of DAMPs such as HMGB1, and promote the polarization of macrophages to M1 macrophages by stimulating TLR4 and other receptors on macrophages. Macrophages polarized to M1 can further secrete ROS to promote liver cell apoptosis and cause liver damage, or they can recruit neutrophils and cause liver damage by secreting TNF-α and other pro-inflammatory factors.

Additionally, KCs can secrete a series of chemokines to attract bone marrow-derived LY6C + monocytes to infiltrate the liver and differentiate into a liver macrophage population with similar functions as those of KCs to affect ischemia-reperfusion injury 17 (Figure 1).

### Polarization of M2 macrophages and liver ischemia-reperfusion injury

Although KCs can aggravate liver ischemia-reperfusion injury, studies have shown that treatment of mice with liposome-encapsulated clodronate and the depletion of KCs in mice results in worsened liver ischemia-reperfusion injury and higher mortality 41. This finding reflects the anti-inflammatory effect of M2 macrophages in liver ischemia-reperfusion injury. 48-72 hours after the occurrence of liver ischemia-reperfusion injury, an increase in M2 type macrophages in the liver can be observed to gradually replace the M1 type macrophages that dominate in the early stage 27. The JAK / STAT signaling pathway plays a key role in the polarization of M2 macrophages. IL-4 and IL-13 can bind with membrane receptors and activate receptor-coupled JAK kinase. IL-4 and IL-13 receptors share a common alpha chain, but they have different subunits 42. Activated JAK kinase can recruit the main members of the IRS (insulin receptor substrates) and form complexes with it, thereby activating the GRB2 (growth factor receptor binding protein 2) and PI3K, recruiting and phosphorylating STAT6 in the nucleus and binding to KLF-4 and PPAR-γ, and promoting M2 polarization by initiating gene transcription. SOCS is an important regulator in the JAK / STAT signaling pathway. The activation of SOCS1 and the inhibition of SOCS3 in the pathway promote the polarization of M2 macrophages 33 IL-4 and IL-13 regulates the program via STAT6 controlled by PPAR-γ and the co-activator protein PGC-1β. PPAR-γ is not necessary to induce M2 macrophages, but is necessary to maintain M2 macrophages 43. Experiments have shown that in mouse models, PPAR-γ agonists significantly improved liver ischemia-reperfusion injury in mice, significantly decreased serum AST compared to that of the control group, decreased the proportion of M1 macrophages in the liver and increased the proportion of M2 macrophages, suggesting that PPAR-γ plays a role in polarizing M2 macrophages and improving liver ischemia-reperfusion 44.

In addition to IL-4 and IL-13, IL-10 is one of the main anti-inflammatory factors released by M2 macrophages, can further promote the M2 polarization of macrophages by activating STAT3 and modulating the effects of IL-4 and IL-13 and IFN-γ 21.

In other disease models, Th2 cells can release IL-33 and IL-25 and enhance M2 macrophages induction 45. IL-21 amplified M2 macrophages by driving IL-4R expression 22. IL-33, which product by a Th2 cell, binds to ST2L, a subunit of the IL-33 receptor, and enhance Th2 cytokine production and M2 macrophages induction 46.

Polarized M2 macrophages can secrete anti-inflammatory factors such as IL-10 and TNF-β to reduce liver ischemia-reperfusion injury. Among them, IL-10 can inhibit the activation of NF-κB and strongly inhibit a series of pro-inflammatory factors such as TNF-α, IL-1β, IFN-γ, IL-2 and macrophage inflammatory protein-2 (MIP-2) secretion 47. Moreover, IL-10 can also inhibit the expression of TNF-α and cell surface adhesion molecules such as E-selectin and ICAM-1 which require activation by TNF-α, thereby reducing liver ischemia-reperfusion injury 48. It has been found that IL-10 can also inhibit TLR4 and inhibit cell death in experimental animals overexpressing IL-10 treated with adenovirus 49. IL-10 can also reduce inflammation-related damage by up-regulating the level of heme oxygenase-1 (HO-1) The heme oxygenase system is the rate-limiting step in the conversion of heme into ferrous iron (Fe^2+^), biliverdin (BV) and carbon monoxide (CO). HO-1 is a heat shock protein-32 induced under IR-stress, is an essential component of the cytoprotective mechanism in stressed livers can be secreted by KCs and can stimulate M2 polarization of macrophages 49-51.

Studies have shown that Kelch-like ECH-associated protein 1 (Keap1)/nuclear factor erythroid 2-related factor 2 (Nrf2)/antioxidant response element (ARE) pathway play a key role in regulating HO-1 levels. Under normal conditions, Nrf2 is sequestered in the cytoplasm by Keap1 and is degraded by the ubiquitin-proteasome pathway. However, under certain conditions, Nrf2 can dissociate from Keap1 and translocate to the nucleus to bind to ARE and up-regulate the transcription of a series of antioxidants and anti-apoptotic proteins including HO-1 52, 53.

**Figure 2:**
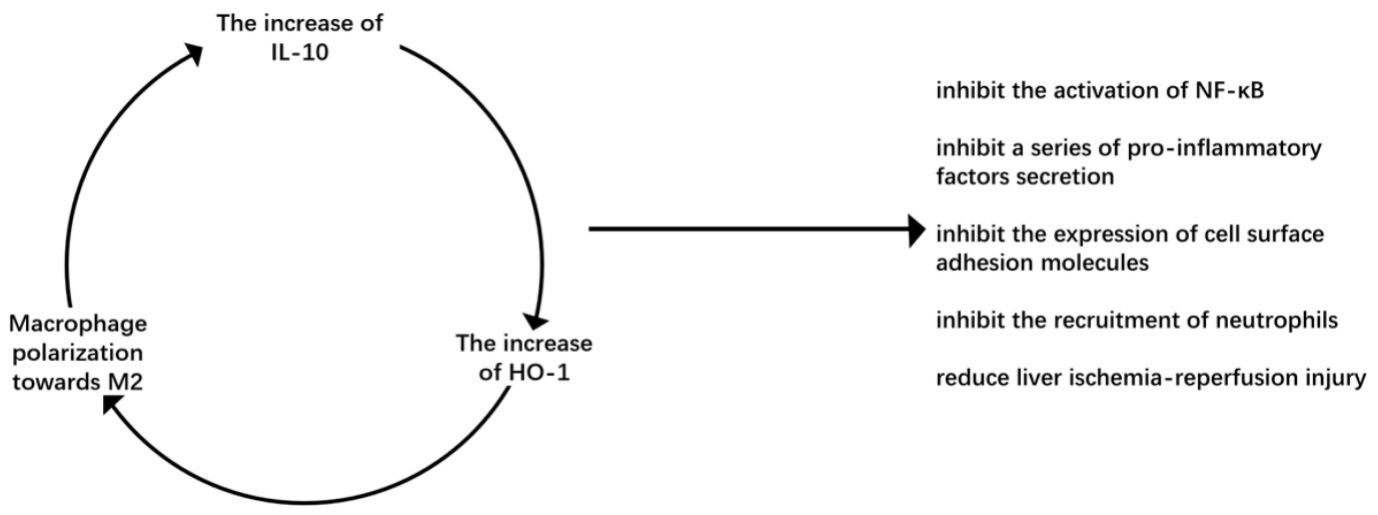
Virtuous circulation of M2 macrophage polarization. IL-10 is the main anti-inflammatory factor secreted by M2 macrophages. It can up-regulate HO-1, an important molecule in the process of macrophages polarizing to M2, to promote macrophages to polarize M2 macrophages. It forms a virtuous cycle to inhibit liver ischemia-reperfusion injury.

It has been found in animal models of warm ischemia-reperfusion and orthotopic liver transplantation that macrophages overexpressing HO-1 can inhibit the recruitment of neutrophils and the expression of proinflammatory factors, reduce liver ischemia-reperfusion injury, and increase the expression of IL-10 to reduce cell death 54. M2-type macrophages secrete IL-10, improve liver ischemia-reperfusion injury and up-regulate the level of HO-1 to further promote M2 macrophage polarization, forming a beneficial cycle that can repair damage (Figure 2).

In addition, in vitro coculture of mesenchymal stem cells (MSCs) with macrophages has shown that MSCs induce M2 polarization. Mechanistically, exosomes derived from MSCs induce macrophage M2 polarization and depletion of exosomes from MSCs reduces the M2 phenotype of macrophages. This finding suggests the potential clinical applications of mesenchymal stem cell transplantation 55.

The intestinal microbiota has also been proven to promote the polarization of M2 macrophages by secreting metastasis-related secretory protein cathepsin K (CTSK), CTSK bind to toll-like receptor 4 (TLR4) to promote the M2 polarization of tumor-associated macrophages via an mTOR-dependent pathway and stimulate tumor-associated macrophages to secrete IL-10 and IL-17 56.

### Prospective application of macrophage polarization in IRI management

IRI is very unfavorable factor in the prognosis of patients during clinical partial hepatectomy and liver transplantation. Preventing IRI is a common concern of many clinicians.

Although there are a lot of studies on the complex signaling pathways of liver macrophages, drugs such as tauroursodeoxycholic acid, ketamine, tanshinone IIA, etc. can selectively act on liver macrophages and improve liver IRI 57-59. However, the research is limited to animal experiments, the feasibility of clinical application is still uncertain, and we need to develop more effective and feasible treatments for IRI.

Using drugs to polarize liver macrophages to M2 type can play a role in improving liver ischemia-reperfusion injury. In addition to the HO-1 and PPAR-γ agonists mentioned above, curcumin, macrophage-targeting fasudil, Resolvin D1 and p300/CBP inhibitor A-485^57^-^60^ can promote M2 polarization of liver macrophages, ameliorate liver IRI, and achieve relatively satisfactory results. These drugs show the excellent effect of treating IRI in different aspects. Fasudil carried liposomes selectively inhibited the expression of ROCK-Ⅱ (Rho-associated protein kinase-Ⅱ) in KCs and monocytes, induced M2 to M1 transformation of macrophages, and prevented the severe hypotension caused by side effects of systematic use of ROCK inhibitor 58. Resolvin D1 promotes the polarization of M2 macrophages and their efferocytosis by activating ALX / FPR2, which in turn protects IRI^57^. Curcumin and P300 / CBP inhibitor A-485 promotes the polarization of macrophages to M2, alleviate the pathological abnormalities of liver tissues, reduces the level of plasma transaminase, and thus alleviate IRI 60. A-485 significantly inhibits H3K27ac/H3K18ac at promoter regions of these crucial inflammatory genes and Curcumin depressed the function of KCs via down-regulating the nuclear factor κb (NF-κb) signaling pathway by stimulating peroxisome proliferator-activated receptor γ (PPARγ) 57, 60. In addition, there are many sites with great therapeutic potential that are worth exploring. Protein interacting with C kinase 1 (PICK1) is a scaffolding protein which be over-expressed can suppress M1 polarization by inhibiting NF-κB activity, and promote M2 polarization by activating STAT6 61. Experiments have shown that hyperglycemia and endoplasmic reticulum stress can cause macrophages to polarize to M1-type during liver ischemia-reperfusion, thereby aggravating liver ischemia-reperfusion injury 62.

Compared with drugs that block the IRI signal pathway, these drugs have better efficacy and can reduce the side effects caused by blocking the signal pathway. This suggests that regulating liver macrophage polarization is an effective method to improve ischemia-reperfusion injury after liver surgery in the future.

Epigenetics and non-coding Mi-RNA also shows great potential in treating IRI. MicroRNA-155 deficiency in KCs can ameliorates liver ischemia-reperfusion injury by regulating the activation and inflammatory response, as well as modifying the polarization of KCs. MicroRNA-155 deficiency in KCs reduces the expression of CD80, CD86 and major histocompatibility complex class II, thereby promoting the differentiation of KCs to M2-type, inhibiting the secretion of pro-inflammatory factors and promoting the secretion of IL-10 63. lncRNA-MM2P has been shown to activate M2 macrophage polarization and inhibit M1 macrophage polarization 35. IncRNA has been shown to play a role in many celluar and developmental processes, including cell proliferation, differentiation and apoptosis, but no previous studies have suggested the role of IncRNA in the differentiation of macrophages into M2-type 64. It was found that IncRNA-MM2P is the only IncRNA in 25 types of IncRNA which can maintain the activation of STAT6, thereby regulating the differentiation of macrophages into M2-type 35. These all suggest the effectiveness of the treatment of IRI aiming at macrophage polarization.

Specific preservation techniques of the liver in liver transplantation can also reduce the IRI of the graft. The α-ketoglutarate (αKG) inhibits the differentiation of KCs into M1 through the glutamine catabolism process, thereby reducing the IRI of the graft. The donor liver can be perfused with DM-αKG (a cell-permeable analog of αKG) or BPTES (an inhibitor of glutaminase 1) through the portal vein during cryopreservation. Compared with the control group, the DM-αKG perfusion group suppressed NF-κB activity, up-regulated the expression of p-GSK3β and SOCS1 in KCs, and shifted macrophages to M2-type, inhibited the pro-inflammatory factors in the serum and promote the secretion of IL-10^65^. Vascular endothelial growth factor receptor-3 (VEGFR-3)/vascular endothelial growth factor-C (VEGF-C) signaling is also believed to inhibit the activation of KCs and thereby inhibit the IRI of graft. Similarly, the donor liver can be perfused with VEGF-C through the portal vein during cryopreservation. RT-PCR, immunofluorescence and western blot techniques suggest that VEGF-C can shift the M1/M2 balance toward an anti-inflammatory profile by up-regulating SOCS-1 and p-GSK3β in KCs 66 (Table 2).

## Conclusion

IRI is an influential factor in the prognosis of patients during partial liver resection and liver transplantation. Preventing and treating IRI is a common concern of many clinicians.

In the initial stages of liver ischemia-reperfusion injury, hypoxia and concomitant metabolic disorders lead to the initial death of hepatocyte and release dangerous signaling molecules such as DAMPs, ROS, and DNA fragments. In late stage of liver reperfusion injury, liver immune cells are activated and recruit more immune cells by producing a series of proinflammatory factors 47. The polarization of liver macrophages plays a key role in IRI. In the early stage of IRI, liver macrophages are mainly M1 type to promote the occurrence of inflammation, and in the later stage of IRI liver macrophages are mainly the M2 type and promote tissue repair.

Experiments have shown that hyperglycemia and endoplasmic reticulum stress can cause macrophages to polarize to the M1-type during liver ischemia-reperfusion, thereby aggravating liver ischemia-reperfusion injury, and microRNA-155 deficiency in KCs can ameliorate liver ischemia-reperfusion injury by regulating the activation and inflammatory response, as well as modifying the polarization of KCs 62, 63, 67.

IRI is an influencing factor that is very unfavorable to the prognosis of patients during clinical partial hepatectomy and liver transplantation. Preventing IRI is a common concern of many clinicians. Polarizing M1 type macrophages to M2 type macrophages shows great potential for the treatment of IRI, and has been verified in clinical studies.

## Figures and Tables

**Table 1:** Macrophage phenotypes and functions

Phenotypes	Stimuli	Surface markers	Secretion	Function
M1	IFN-γ, TNFα, LPS, TLR ligands	CD80, CD86, CD68, MHC-II, IL-1R, TLR-2, TLR-4, IL-10 low, IL-12 high	TNF-a, IL-6, TNF-α, IL-1, IL-12, IL-15, IL-18, CCL2, CCL3, CCL5, CXCL9, CXCL10, CXCL11, CXCL15, CXCL20, ROS, iNOS, NO	Pro-inflammatory Th1 immune response, tumor resistance
M2a	IL-4, IL-13	CCR2, IL-1R, MMR/CD206	CCL13, CCL14, CCL22, CCL23, CCL24, TGF-β, PDGF, MMP-9, MMP-12, Arginase-1	Anti-inflammatory Th2 immune response, tissue remodeling
M2b	Immune Complexes, TLR ligands, IL-1β	CD80, CD86	IL-1, IL-6, IL-10, IL-12, CCL1	Pro-fibrotic, immunoregulation
M2c	IL-10, TGF-β,glucocorticoids	CD150,CD163,IL-1R, MMR/CD206	IL-10, TGF-β, Arginase-1, CCL16, CCL18, CXCL13	Immune suppression, tissue repair

**Table 2:** Prospective application of macrophage polarization in IRI management

Methods		Effect on molecular pathways	Effect on IRI
Fasudil carried liposomes		inhibiting the expression of ROCK-II in KCs	shifting the M1/M2 balance toward an anti-inflammatory profile; inhibiting the secretion of pro-inflammatory factors and promoting the secretion of anti-inflammatory factors; alleviating liver ischemia-reperfusion injury
P300 / CBP inhibitor A-485		inhibiting H3K27ac/H3K18ac at promoter regions of pivotal inflammatory genes	
Curcumin		down-regulating NF-κb signaling pathway by stimulating PPARγ	
Epigenetics and non-coding Mi-RNA	MicroRNA-155	reducing the expression of CD80, CD86 and major histocompatibility complex class II	
	IncRNA	maintaining the activation of STAT6	
preservation techniques of the liver	αKG	up-regulating the expression of p-GSK3β and SOCS1	
	VEGF-C	up-regulating the expression of p-GSK3β and SOCS1	
